# RNA-seq coupling two different methods of castration reveals new insights into androgen deficiency-caused degeneration of submaxillary gland in male Sprague Dawley rats

**DOI:** 10.1186/s12864-022-08521-9

**Published:** 2022-04-07

**Authors:** Xingfa Han, Xue Xia, Yong Zhuo, Lun Hua, Guozhi Yu, Guixian Bu, Xiaohan Cao, XiaoGang Du, Qiuxia Liang, Xianyin Zeng, Fengyan Meng

**Affiliations:** 1 Isotope Research Lab, Biological Engineering and Application Biology Department, Sichuan Agricultural University, Ya’an 625014, China; 2 Key Laboratory for Animal Disease-Resistant Nutrition of the Ministry of Education of China, Animal Nutrition Institute, Sichuan Agricultural University, Chengdu, 611130 China

**Keywords:** Immunocastration, Surgical castration, Submaxillary gland, Degeneration, Transcriptomics, Rat

## Abstract

**Background:**

Salivary gland (SMG) degeneration and dysfunction are common symptoms that occur after sex hormone deprivation, but the underlying mechanisms remain largely unknown. Additionally, immunocastration, which causes drop of sex hormones, has been developed as an alternative to surgical castration, however whether it exerts similar effects as surgical castration on the salivary glands is unknown. Through histological and RNA-seq analysis, we assessed changes in morphology and transcriptome of SMG in response to immunocastration (IM) versus surgical castration (bilateral orchiectomy, ORC).

**Results:**

Compared to entire males (EM), ORC caused severe degeneration of SMG in rats, as evidenced by both decreased (*P* < 0.01) SMG weight and organ index, and by decreased (*P* < 0.01) quantity of SMG acini and ducts. IM had minimal effects (*P* > 0.05) on SMG weight and organ index, but it still caused degeneration (*P* < 0.05) of the acini and ducts. Even though, the quantity of both SMG acini and ducts was much higher (*P* < 0.001) in IM than in ORC. Functional enrichment analysis of the common regulated genes by ORC/IM revealed disrupted epithelial cell development, angiogenesis, anatomical structure morphogenesis and enhanced cell death are associated with SMG degeneration in deprivation of androgens. Integrated data analysis shown that there existed a selective hyperfunction of SMG ribosome and mitochondrion in ORC but not in IM, which might be associated with more severe degeneration of SMG in ORC than in IM.

**Conclusions:**

Our findings suggested that both surgical castration and immunocastration caused SMG degeneration by disrupting epithelial cell development, angiogenesis, anatomical structure morphogenesis and enhancing cell death. But, surgical castration selectively induced hyperfunction of SMG ribosome and mitochondrion, thus causing more severe degeneration of SMG than immunocastration.

**Supplementary Information:**

The online version contains supplementary material available at 10.1186/s12864-022-08521-9.

## Background

The salivary glands are essential structures of the mouth, with the main role of secreting saliva to maintain oral cavity health and functionality. There are three major salivary glands, i.e., the parotid, submandibular (submaxillary) and sublingual glands. Within those salivary glands, saliva is secreted by acinar cells, which are categorized into mucous and serous acinar cells. Most of the acinar cells in parotid glands are serous, secreting α-amylase-rich saliva, and those of the sublingual glands are mucous, secreting a viscous solution rich in mucins [1]. While, the submandibular glands (SMG) are composed by a mixed population of acini with approximately 90% serous cells and 10% mucous cells [1]. The three major salivary glands account for approximately 95% of salivary secretion in the oral cavity [2].

Saliva exerts a crucial role in initial breakdown of foods, mastication, swallowing, speech, and taste [1]. Hypo-salivation caused by dysfunction of salivary glands commonly induces the poor oral health and dry mouth symptoms. Specifically, it compromises the clearance of food debris and bacteria from the oral cavity, causes mastication, swallowing and speech difficulties, affects taste, exacerbates dental caries and increases oral infections [3].

Sex steroid hormones seem to play a great role in the physiology of salivary glands. Previous studies have reported degeneration of the submaxillary gland tubules after hypophysectomy and recovery after administration of testosterone in male rats [4]. Similarly, ovariectimized rodents and menopausal women also shown salivary gland degeneration and dysfunction, characterized by a reduction in the quantity of salivary acini and ducts [5–7]. It is clear that dysfunction of salivary glands secondary to deficiency of sex steroids, like menopause in women usually causes a significant decrease in salivary flow, resulting in hypo-salivation and xerostomia [8]. However, the mechanisms by which deficiency of sex steroids cause degeneration and dysfunction of salivary glands still remain largely unknown. Additionally, to date, studies on the relationship between sex steroids and the functionality of salivary glands are mainly focused on females, especially on menopausal women [5–7], few studies have been carried out on males.

Active immunization against gonadotropin-releasing hormone (GnRH), which is also called as immunocastration, has been developed as an alternative to surgical castration in farm and companion animal species for avoiding surgical castration-caused health, welfare and/or productivity drawbacks [9]. And, it is also applied as a means for treating sex hormone-dependent diseases, e.g., prostate cancer in men [10]. Very like surgical castration, immunocastration causes a large drop or even deficiency of sex hormones across mammalian species [9, 11–13]. However, whether immunocastration exerts a profound effect, just like surgical castration, on the morphology and physiology of salivary glands is totally unknown.

In the present study, using a rat model, we investigated the effects of testicular steroid deprivation and also compared effects of immunocastration versus surgical castration on the morphology and function of submaxillary gland (SMG) in males. Especially, through RNA-sequencing (RNA-seq) technique, we profiled the global gene expression of SMG in response to the two different methods of castration and provided new insights into the role of reproductive endocrinology in regulating salivary gland physiology and function.

## Results

### Anti-GnRH vaccination triggered a good immunocastration response in all immunized male rats

**Fig. 1:**
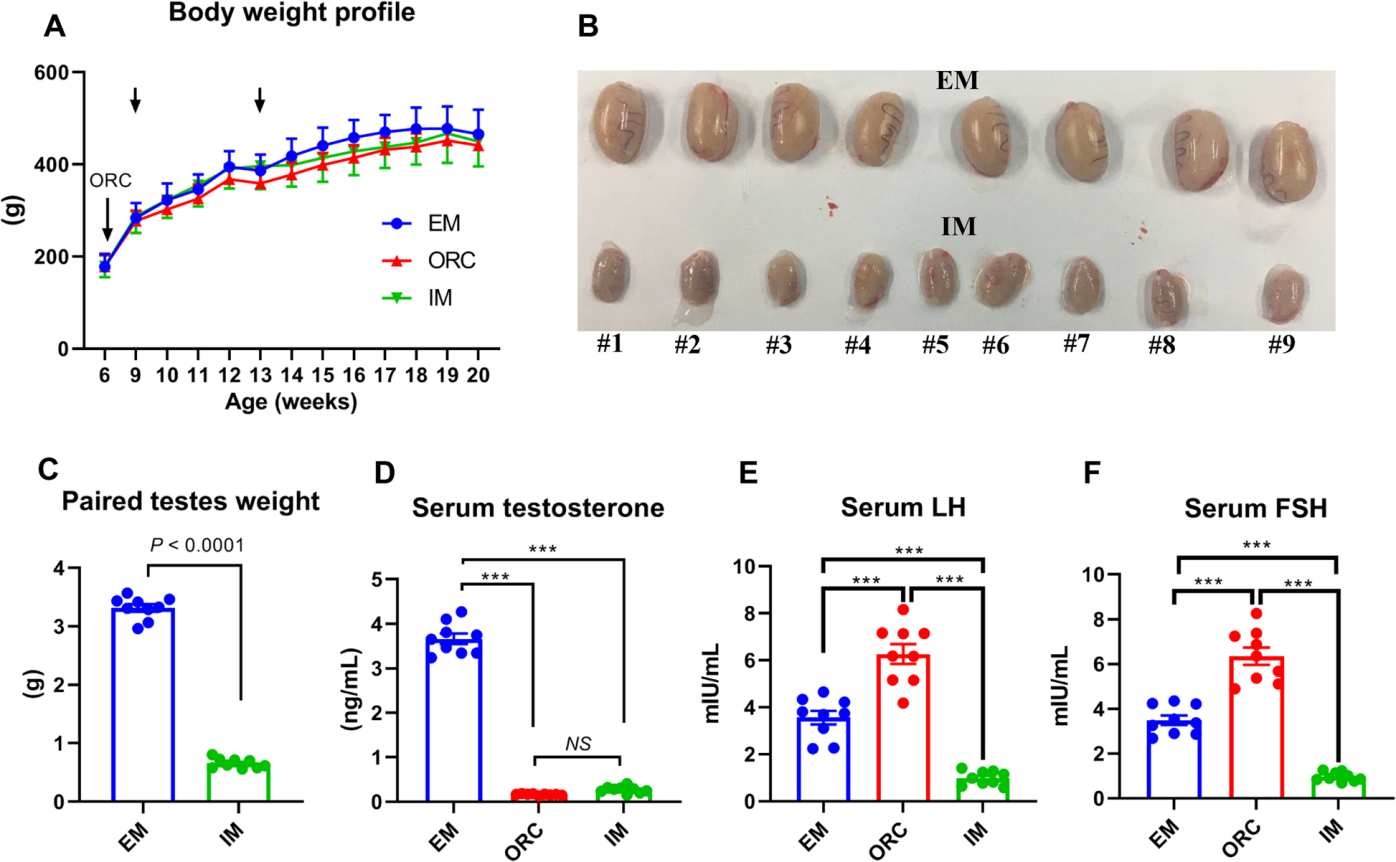
Anti-GnRH immunization induced a good immunocastrative effects in all the immunized rats. (**A**) Body weight profile of rats across the whole experimental periods. (**B**) Testes from all the immunized rats were obviously atrophied. (**C**) Paired testes weight at decapitation was largely declined in immunized rats. (**D-F**) Serum testosterone, LH and FSH concentrations in rats at decapitation. Data are expressed as mean ± SEM. ^***^P < 0.001

Compared to EM, neither ORC nor IM had obvious effects on the body weight profile of rats (*P* > 0.05; Fig. 1A). After two doses of GnRH vaccine injection, all the 9 immunized rats shown a substantial decrease (*P* < 0.001; Fig. 1B and C) in testes weight. At decapitation, the average testes weight of immunized rats was reduced by 80% compared to that of EM (Fig. 1C). Resultantly, serum concentrations of testosterone were reduced (*P* < 0.001; Fig. 1D) to undetectable levels, indicating a very good immunocastration efficacy in all the immunized rats. Compared to EM, both serum LH and FSH concentrations were substantially increased in ORC (*P* < 0.001), but reduced (*P* < 0.001) in IM (Fig. 1E and F).

### Differential efficacy of ORC versus IM in causing degeneration of SMG in male rats

**Fig. 2:**
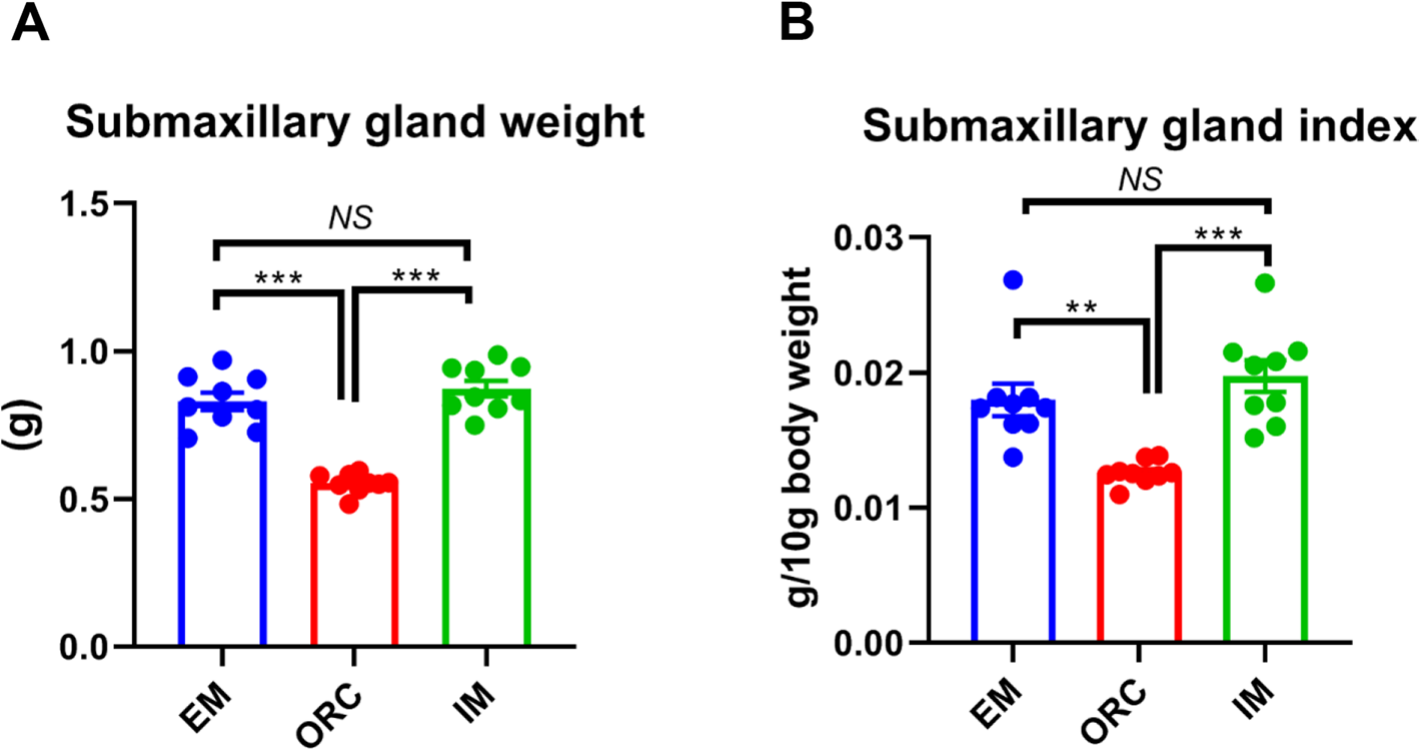
Surgical castration but not immunocastration caused a dramatic degeneration of submaxillary gland in male rats. (**A**) Submaxillary gland weight. (**B**) Submaxillary gland index. Data are expressed as mean ± SEM. ^***^P < 0.001; ^**^P < 0.01; *NS*, not significant

Compared to EM, ORC caused a substantial degeneration of SMG. Both the weight and organ index of SMG were markedly decreased (*P* < 0.01) in rats following ORC (Fig. 2A-B). But, there was no obvious difference in either SMG weight or organ index between EM and IM (*P* > 0.05). And both the weight and organ index of SMG in IM were higher (*P* < 0.001) than in ORC.

**Fig. 3:**
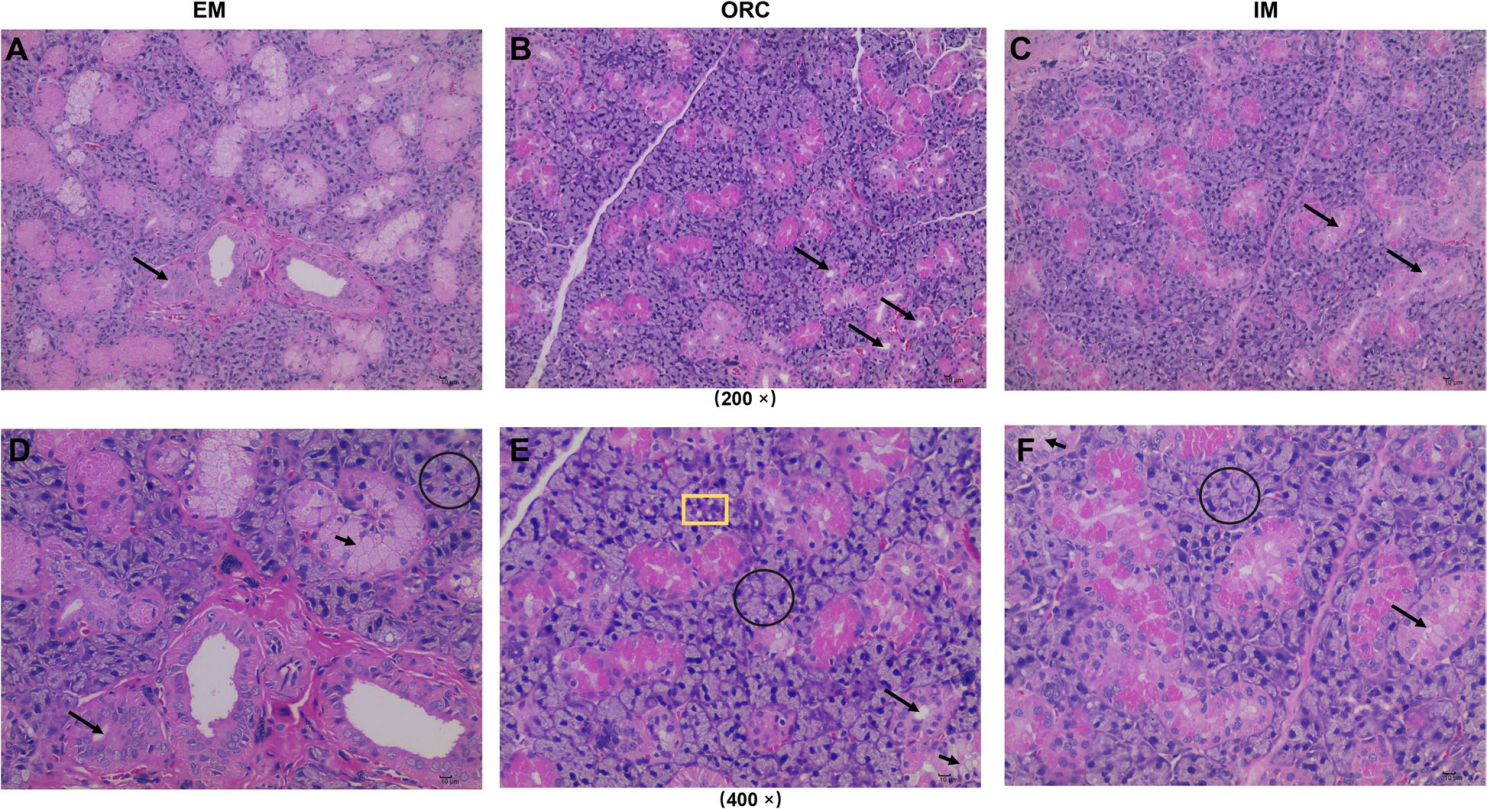
Histological analysis of submaxillary gland in male rats. (**A-D**) Entire male rats exhibited predominantly serous acini (circle), lower quantity of mucous acini (short arrow) and bigger ducts (long arrow). (**B-E**) Bilateral orchiectomy caused atrophic ducts (long arrow) and increased lipid vacuoles (yellow square). (**C**-**F**) Immunocastration also caused moderate atrophy of ducts in SMG. Scale bars = 10 μM. A-C: original magnification 200 × ; D-E: original magnification 400 × ; Tissues were stained with hematoxylin–eosin

**Fig. 4:**
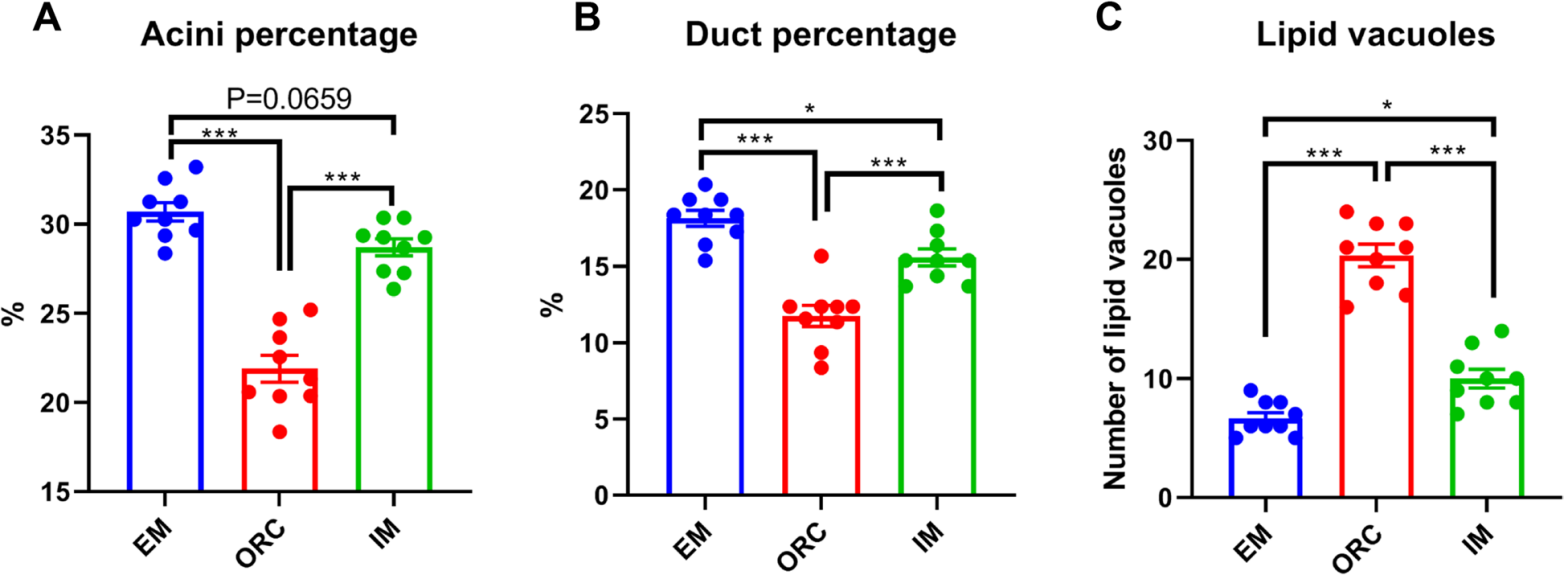
Statistical results of the percentage of acini and ducts as well as number of lipid vacuoles in SMG of male rats. Observe the decreased percentage of acini (**A**) and ducts (**B**) and increased number of lipid vacuoles (**C**) in SMG in ORC rats. While, IM rats only shown a moderate decrease in percentage of acini and ducts, and a moderate increase in lipid vacuoles in SMG. Data are expressed as mean ± SEM. ^***^P < 0.001; ^*^P < 0.05

Histological analysis shown that the SMG exhibited predominantly serous acini and a lower quantity of mucous acini, and an obvious degeneration of SMG acini and ducts in ORC (Fig. 3A and B). Statistical analysis indicated that ORC substantially decreased (*P* < 0.001) the percentage of both SMG acini and ducts (Fig. 4A and B), indicating a substantial degenerative alteration in the histological structure of SMG. Likewise, IM also moderately reduced the percentage of SMG acini (*P* = 0.0659) and ducts (*P* < 0.05), but both of which were still much higher (*P* < 0.001) than those in ORC (Fig. 3B and C; Fig. 4A and B).

Compared to EM, there was a substantial increase (*P* < 0.001) in lipid vacuoles in SMG in rats following ORC (Figs. 3E and 4C). Although, lipid deposition was also increased (*P* < 0.05) in SMG of rats following IM, it was markedly lower than that in ORC (*P* < 0.001; Figs. 3F and 4C).

### Transcriptomic changes of SMG in response to ORC versus IM

**Fig. 5:**
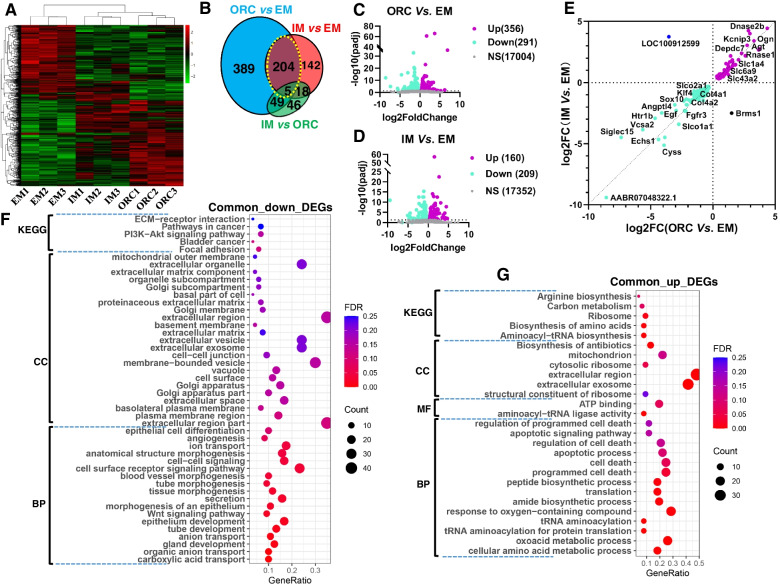
Transcriptomic analysis reveals the cause of SMG degeneration in androgen deprivation. (**A**) Hierarchical clustering analysis of the differentially expressed genes based on the z-score of their FPKM value. (**B**) Venn diagram of differentially expressed genes (DEGs) among groups. (**C**) Differentially expressed genes (DEGs) between ORC and EM. (**D**) Differentially expressed genes (DEGs) between IM and EM. (**E**) The common regulated genes in SMG by ORC and IM. (**F-G**) Functional enrichment analysis of the common downregulated and common upregulated DEGs, respectively using DAVID (6.8). Up, upregulated; Down, downregulated; NS, non-significant; MF, Molecular function; CC, Cellular component; BP, Biological process; KEGG, Kyoto encyclopedia of genes and genomes; EM, entire males; ORC, bilateral orchiectomy; IM, immunocastration

To gain a better understanding of the mechanisms of testicular steroids deficiency on SMG physiology, we assessed the transcriptomic changes of SMG in response to ORC versus IM by RNA-seq (*n* = 3). Hierarchical clustering comparing patterns of gene expression indicated a striking separation in SMG among groups (Fig. 5A), revealing a distinct functional dissociation in SMG between ORC/IM and EM, and between IM and ORC. Even though, SMG samples from IM were still clustered closer to those from ORC than from EM, suggesting there exist some common causes to SMG degeneration between ORC and IM.

Pairwise comparison among groups revealed 851 differentially expressed genes (DEGs, Fig. 5B; see Materials and Methods for criteria). Of those, 647 DEGs were identified between ORC and EM, of which 356 (55%) were upregulated and 291 (45%) were downregulated in ORC (Fig. 5C; Table S2). And, 369 DEGs were identified between IM and EM, of which 160 (44.6%) were upregulated and 209 (55.4%) were downregulated in IM (Fig. 5D; Table S2). There were 209 common DEGs between ORC and EM, and between IM and EM (Fig. 5B, yellow circle; Table S3). Except *Brms1* and *LOC100912599*, all other 207 common DEGs showed same direction of changes (up- or downregulation) comparing EM (Fig. 5E). Of those common DEGs, 126 (61%) were common downregulated and 83 (39%) were common upregulated in ORC/IM (Table S3).

Functional enrichment analysis using DAVID (V6.8) shown that the common downregulated DEGs in ORC/IM were predominantly annotated into biological process (BP) of epithelial cell differentiation, angiogenesis, ion transport, anatomical structure morphogenesis, secretion, gland/tube development (Fig. 5F). Dysfunction of these biological processes, especially dysfunction of epithelial cell development, angiogenesis and anatomical structure morphogenesis should cause SMG degeneration. Cellular component (CC) analysis shown that they were mainly annotated into membrane-bounded vesicle, extracellular exosome, golgi apparatus, etc. (Fig. 5F), suggesting a declined saliva secretion of SMG after androgen deprivation. And, KEGG pathway analysis highlighted PI3K-Akt signaling pathway, focal adhesion and ECM-receptor interaction (Fig. 5F) may play great roles in mediating androgen deprivation-induced SMG degeneration. While, the common upregulated DEGs were predominantly annotated into BP of oxoacid metabolic process, amide biosynthetic process, translation, cell death, apoptotic process/signaling pathway, regulation of programmed cell death etc., into MF of aminoacyl-tRNA ligase activity, ATP binding, etc., into CC of extracellular exosome, cytosolic ribosome, mitochondrion, into KEGG pathways of antibiotics and amino acids biosynthesis, carbon metabolism, etc. (Fig. 5G). Obviously, increased cell apoptosis/death also should be another important cause of SMG degeneration in androgen deprivation.

### Identification of key candidate genes that mediate SMG degeneration in response to androgen deprivation

**Fig. 6:**
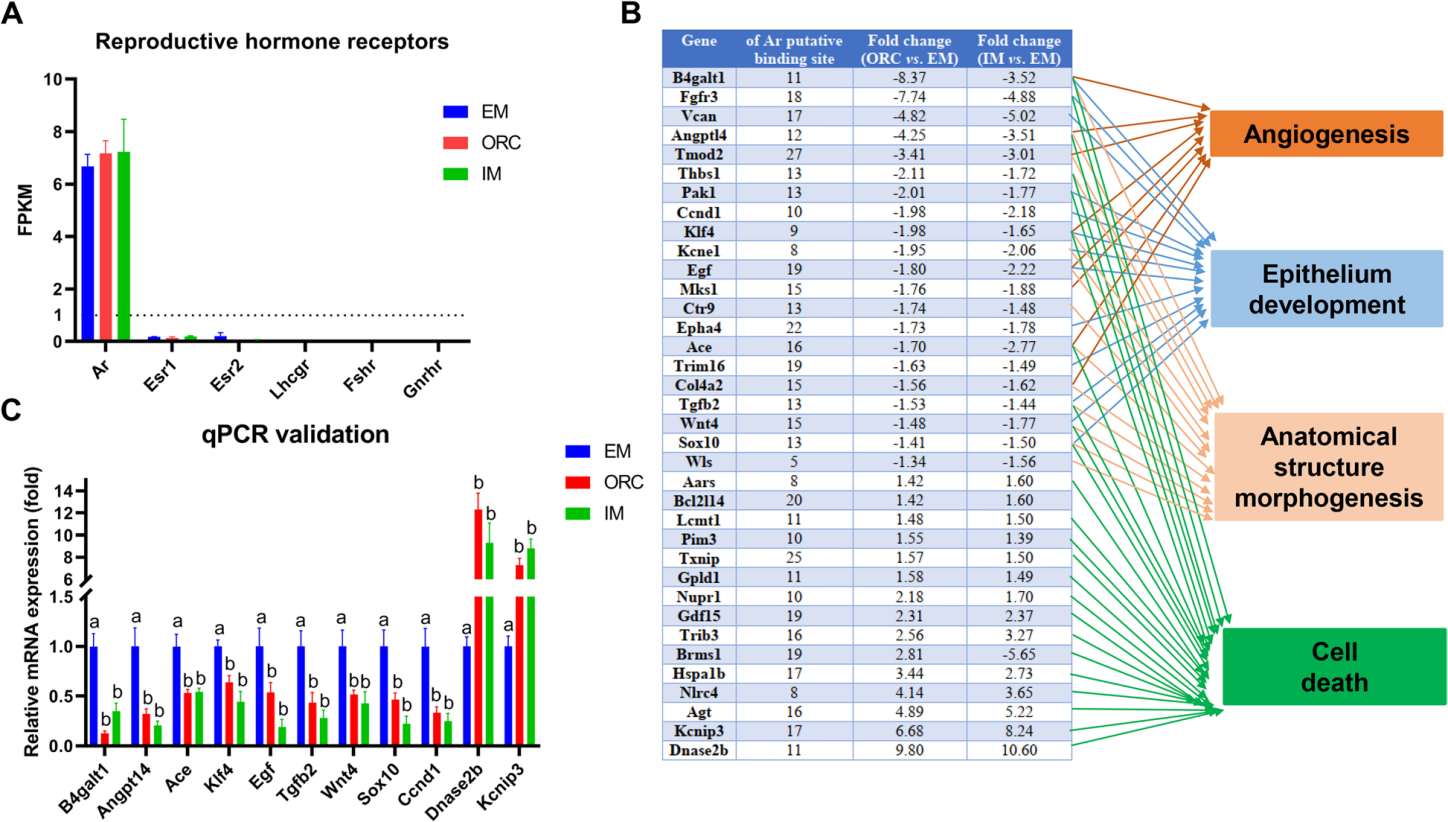
Key candidate genes mediating androgen deprivation-caused degeneration of SMG. (**A**) Expression of receptors for reproductive hormones in SMG. (**B**) Number of putative androgen receptor (AR) binding site in promoter regions of common downregulated genes involved in epithelium development, anatomical structure morphogenesis, angiogenesis and cell apoptosis. De novo motif analysis was performed by Jaspar with 3 kb upstream and 500 downstream (promotor) sequence for the genes. 80% was used as the relative profile score threshold. Note common DEGs shown herein are the ones with fold change ≥ 1.5 at least in one pairwise comparison. Functional enrichment analysis was analyzed using DAVID (6.8). (**C**) qPCR validation of the key candidate genes mediating androgen deprivation-caused degeneration of SMG. Data are expressed as mean ± SEM; different superscripts indicate *P* < 0.05. *B4galt1*, beta-1,4-galactosyltransferase 1; *Angptl4*, angiopoietin-like 4; *Ace*, angiotensin I converting enzyme; *Klf4*, Kruppel like factor 4; *Egf*, epidermal growth factor; *Tgfb2*, transforming growth factor, beta 2; *Wnt4*, Wnt family member 4; *Sox10*, SRY-box transcription factor 10; *Ccnd1*, cyclin D1; *Dnase2b*, deoxyribonuclease 2 beta; *Kcnip3*, potassium voltage-gated channel interacting protein 3

To identify candidate genes that mediate SMG degeneration in response to androgen deprivation, we checked the expression of reproductive hormone receptors in SMG across groups. Using FPKM ≥ 1 in at least one of the sample replicates as a stringent cutoff for gene expression [14], we found that SMG expresses androgen receptor (*Ar*) but not expresses receptors for estrogens, gonadotropins (LH and FSH) or gonadotropin-releasing hormone (GnRH) (Fig. 6A), suggesting androgen deprivation is of high possibility to be the main or even the only cause of SMG degeneration following surgical or immunological castration. Given disrupted epithelial cell development, angiogenesis, anatomical structure morphogenesis and enhanced cell apoptosis are directly relevant to SMG tissue structural and morphological remodeling, we selected the common regulated genes by both ORC and IM which are involved in these four functional terms to conduct further analysis. De novo* motif* analysis of these selected genes revealed a high number of putative AR binding sites within their promoter regions (Fig. 6B), suggesting these genes might be the direct targets of androgens in regulating SMG structure and morphology remodeling. Of those important candidate genes, *B4galt1*, *Angpt14*, *Ace*, *Klf4*, *Egf*, *Tgfb2*, *Wnt4* and *Sox10* are simultaneously involved epithelial cell development, angiogenesis, anatomical structure morphogenesis and/or cell apoptosis and their expressions were all significantly downregulated by androgen deprivation (Fig. 6B), thus they might be the key candidate genes that mediate androgen deprivation-induced SMG degeneration. Besides, *Dnase2b* (deoxyribonuclease II beta), an enzyme responsible for nuclear degradation, plays a major role in cellular apoptosis [15]. And, *Kcnip3* (also called *Dream*) enhances cell apoptosis by altering endoplasmic reticulum calcium signaling [16]. Both of the two genes were commonly upregulated to 7 ~ tenfold by androgen deprivation, thus they appear to play key roles in SMG degeneration in rats following ORC/IM through enhancing cell apoptosis. Using RT-qPCR, we validated the expression changes of all these key candidate genes (Fig. 6C).

### Functional enrichment analysis reveals the causes of differential degeneration response of SMG between ORC and IM

**Fig. 7:**
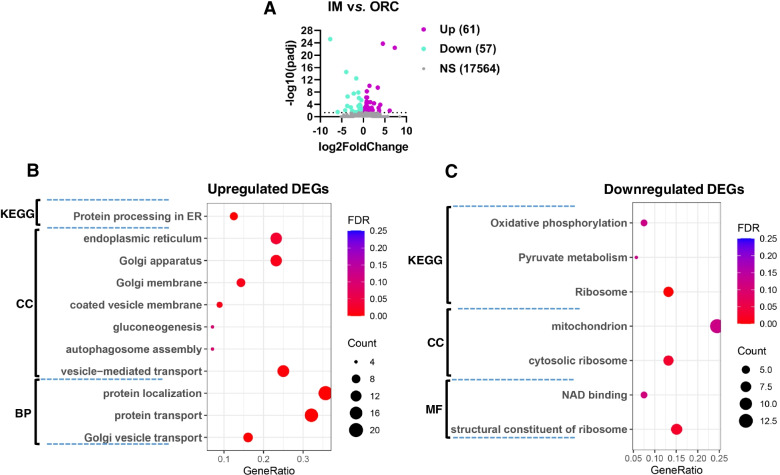
Functional enrichment analysis of differentially expressed genes between IM and ORC. (**A**) Volcano plot of the differentially expressed genes between IM and ORC. (**B-C**) Functional enrichment analysis of the upregulated and downregulated DEGs in IM, respectively using DAVID (6.8). MF, Molecular function; CC, Cellular component; BP, Biological process; KEGG, Kyoto encyclopedia of genes and genomes

Functional enrichment analysis of the DEGs between IM and ORC indicated that the upregulated DEGs in IM were mainly annotated into CC of coated vesicle membrane, golgi apparatus and endoplasmic reticulum, into BP of golgi vesicle transport, protein transport, and into KEGG pathways of protein processing in endoplasmic reticulum (Fig. 7A; Table S4), suggesting a stronger capacity of SMG to secrete saliva in IM than in ORC. While, the downregulated DEGs in IM were mainly annotated into CC of ribosome, mitochondrion, into MF of structural constituent of ribosome, NAD binding, and into KEGG pathways of ribosome and oxidative phosphorylation (Fig. 7B), highlighting a hyperfunction of ribosome and mitochondrion of SMG in ORC than in IM.

**Fig. 8:**
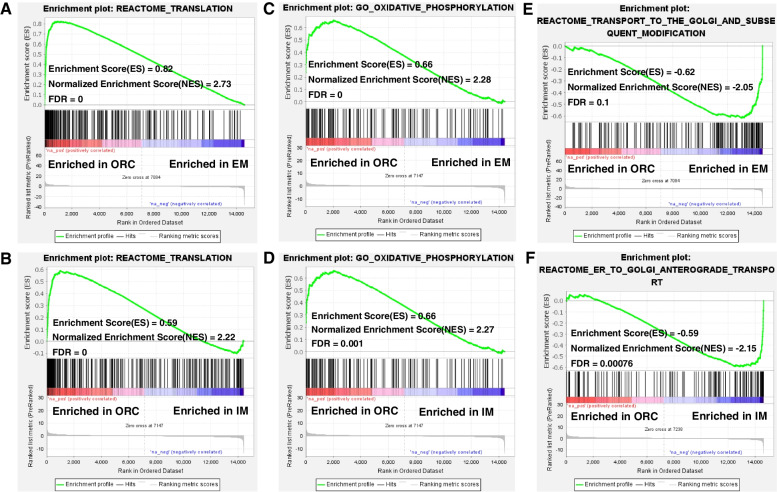
Gene set enrichment analysis highlights hyperfunction of ribosome and mitochondria of SMG in ORC rats. (**A-B**) GSEA of translation in SMG of ORC in relative to EM/IM. (**C-D**) GSEA of oxidative phosphorylation in SMG of ORC in relative to EM/IM. (**E–F**) GSEA of vesicle transport in SMG of ORC in relative to EM/IM

Gene set enrichment analysis (GSEA) using the whole expressed genes were performed. Interestingly, compared to either EM or IM, ORC rats consistently shown a positive enrichment score for protein translation (Fig. 8A and B) and oxidative phosphorylation (Fig. 8C and D), and negative score for vesicle transport (Fig. 8E and F), reaffirming hyperfunction of ribosome and mitochondrion, and lower salivary secretion of SMG in ORC rats than in IM/EM rats.

**Fig. 9:**
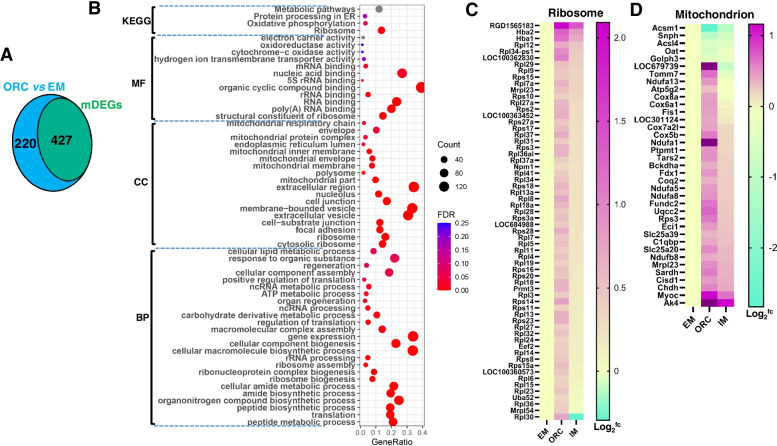
The maintained DEGs and their functional enrichment analysis. (**A**) Venn diagram showing the number of the maintained DEGs (mDEGs). mDEGs refer to as genes whose expressions in SMG were significantly altered after ORC compared to EM, but remained unchanged after IM. (**B**) Functional enrichment analysis of the mDEGs using DAVID (6.8). (**C**) Heatmap of the mDEGs involved in ribosome. (**D**) Heatmap of the mDEGs involved in mitochondrion. MF, Molecular function; CC, Cellular component; BP, Biological process; KEGG, Kyoto encyclopedia of genes and genomes

To further gain insights into the differential SMG degeneration response between ORC and IM, we isolated the genes which were differentially expressed between ORC and EM (adj < 0.05), but remained unchanged between IM and EM. We referred them to as the maintained DEGs (mDEGs). In other words, these mDEGs were selectively affected by ORC but not by IM. Thus, they are directly relevant to the differential SMG degeneration response between IM and ORC. There were 647 DEGs between ORC and EM, of which 427 (66%) were the mDEGs (Fig. 9A; Table S2). Functional enrichment analysis showed that these mDEGs were predominantly annotated into CC of ribosome, mitochondrion, focal adhesion, cell-substrate junction, extracellular vesicle, into MF of structural constituent of ribosome, RNA binding, into BP of translation, ribosome biogenesis, gene expression, cellular component biogenesis, cellular lipid metabolic process, into KEGG pathways of ribosome, oxidative phosphorylation, protein processing in endoplasmic reticulum and metabolic pathways (Fig. 9B). Of 427 mDEGs, 62 mDEGs are associated with ribosome (Fig. 9C) and 37 associated with mitochondrion (Fig. 9D). Almost, all of these mDEGs involved in ribosome and mitochondrion were expressed higher in ORC than in EM/IM (Fig. 9 C and D), emphasizing hyperfunction of ribosome and mitochondrion of SMG in ORC again.

### Integrated network analysis revealed candidate mediators of the differential SMG degeneration response between ORC and IM

**Fig. 10:**
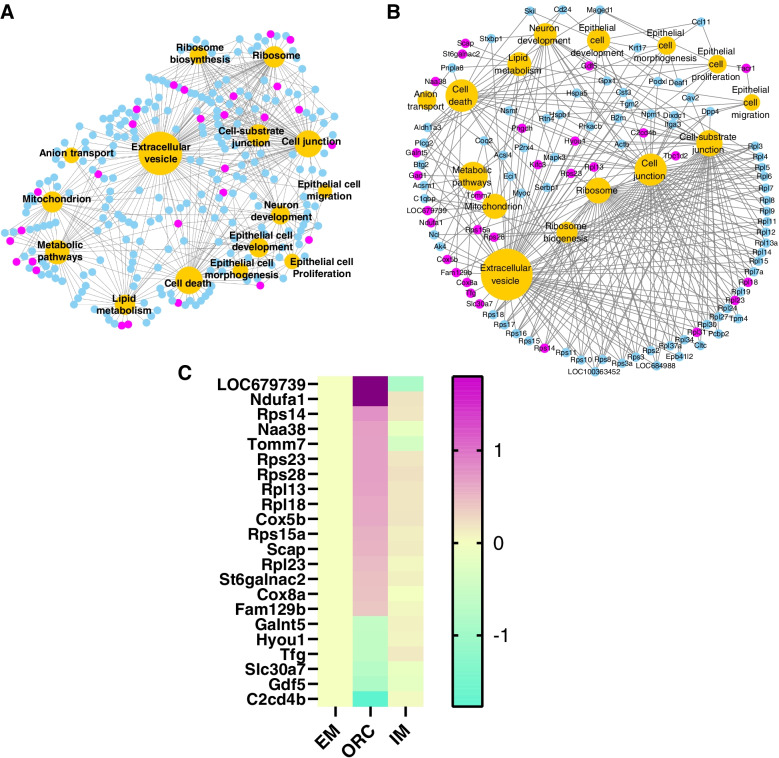
Integrated analysis reveals candidate mediators of the differential SMG degeneration response between ORC and IM. (**A**) Network constructed with functional terms (epithelial cell development/proliferation/morphogenesis, ribosome, mitochondrion, neuron development, anion transport, lipid metabolism, cell junction, and extracellular vesicle) and the mDEGs involved in these terms. (**B**) A subnetwork derived from the mDEGs that were differentially expressed (adj ≤ 0.05) between IM and ORC, and from the mDGEs that are simultaneously involved in three or more functional terms

Oxidative stress and apoptosis have been considered as the mechanisms of dysfunction of salivary gland in women after menopause [3]. Functional enrichment analysis of our SMG transcriptomic data highlighted that epithelial cell development/proliferation/morphogenesis, cell death, neuron development, anion transport, cell junction, extracellular vesicle, etc., might be associated with the degeneration response of SMG in deprivation of androgens (Fig. 5F and G; Table S4). Besides, histology analysis shown that lipid deposition was also an important reason to cause SMG degeneration in deprivation of androgens. Comprehensive analysis suggests that hyperfunction of ribosome and mitochondrion might be associated with the differential degeneration response of SMG between IM and ORC (Fig. 7B and C). We isolated the mDEGs involved in those above terms to construct a network using Cytoscape software (v3.9) (Fig. 10A). We further extracted a subnetwork (Fig. 10B) derived from 1) the mDEGs that were differentially expressed between IM and ORC, and 2) the mDGEs that are simultaneously involved in three or more functional terms. The mDEGs in Fig. 10B thus should play important roles in mediating the differential degeneration response of SMG between IM and ORC. Particularly, 27 genes (Fig. 10C) in this subnetwork are mDEGs and also DEGs between IM and ORC. We consider these 27 genes are the key candidate genes that mediate the differential degeneration response of SMG between IM and ORC. Of these 27 genes, *Naa38* and *Fam129b* is involved in cell death; *Cox5b*, *Ndufa1*, *LOC679739* (Ndufs6) and *Tomm7* are all involved in mitochondrion; *Rps14*, *Rpl23, Rpl13*, *Rpl18*, *Rps15a*, *Rps23* and *Rps28* are all involved in ribosome; *Scap* and *St6galnac2* are both associated with lipid metabolism. Expression of these genes was all upregulated in ORC but kept unchanged or downregulated in IM, in accordance with more severe degeneration of SMG in ORC than in IM (Fig. 10C). While, *Hyou1* is involved in cell junction and extracellular vesicle; *Gdf5* is involved in epithelial cell proliferation; *C2cd4b is* involved in both cell junction and cell-substrate junction; *Tfg* and *Slc30a7* are both involved in extracellular vesicle. These genes were all downregulated in ORC but kept unchanged in IM (Fig. 10C), also in accordance with more severe degeneration of SMG in ORC than in IM.

## Discussion

Increasing studies have evidenced dysfunction of salivary gland after deprivation of sex hormones [2, 3, 17]. Immunocastration, which causes a decrease or even deficiency of sex hormones, has been developed as an alternative to surgical castration [11–14, 18]. However, to the best of our knowledge, so far no studies have been performed to investigate the effects of immunocastration on the salivary glands. In this study, we found that immunocastration had minimal effects on SMG weight or organ index. Even though, the quantity of the acini and ducts of SMD in IM was moderately decreased compared to EM, still showing certain extent of degeneration and dysfunction. In stark contrast, surgical castration caused a substantial degeneration of SMG, as evidenced by both decreased organ weight and index, and by obvious degeneration of the acini and ducts. The percentage of both SMG acini and ducts in ORC was markedly lower than in IM, indicating a more severe degeneration of SMG in ORC than in IM. Dysfunction of salivary glands commonly induces hypo-salivation and causes poor oral health and dry mouth symptoms [7, 8]. In this regard, immunocastration appears to be more superior and suitable than surgical castration to be applied as a castration approach in animal husbandry and companion animal species, or as a treatment for sex hormone-dependent diseases in men.

Several mechanisms such as oxidative stress, apoptosis and ferroptosis have been reported as the mechanisms of degeneration and dysfunction of salivary glands in ovariectomized rodents and postmenopausal women [3, 5]. However, few studies have been performed to elucidate the mechanisms of degeneration and dysfunction of salivary glands in males after deprivation of sex hormones. In this study, we assessed and compared the genome-wide transcriptional profiles of SMG in male rats in response to two different methods of sex hormone deprivation (i.e., immunocastration versus surgical castration). Interestingly, almost all the common regulated DEGs by ORC/IM shown the same direction of changes (up- or down-regulation), suggesting common causes of SMG degeneration in both ORC and IM. Given testicular steroids are consistently decreased, while pituitary LH and FSH undergo opposite changes between ORC and IM [11–13]. Accordingly, we consider that deprivation of testicular steroids should be the main cause of SGM degeneration in rats after both ORC and IM. Indeed, using FPKM = 1 as a stringent criteria for gene expression [14], we only observed *Ar* but not *Fshr*, *Lhcgr*, *Esr1*, *Esr2* or *Gnrhr* expression in SMG in rats, excluding the possible impacts directly from other reproductive hormones. In support of our concepts, previous studies also evidenced that degeneration of SMG caused by hypophysectomy in male rats could be recovered after administration of testosterone [4].

Further functional enrichment analysis shown that the common downregulated DEGs by both ORC and IM were predominantly annotated into epithelial cell differentiation, angiogenesis and anatomical structure morphogenesis, while the common upregulated DEGs were predominantly annotated into cell death. As these four biological processes are all highly relevant to organ/tissue development and morphogenesis, thus they should play great roles in mediating the degeneration and dysfunction of SMG in deprivation of androgens. De novo motif analysis of the common DEGs involved in the above four biological processes revealed a high number of putative androgen receptor (AR) response elements (AREs) within their promoter loci (Fig. 6B), reaffirming these genes may be subject to direct regulation by AR (Fig. 6B). Particularly, the common downregulated DEGs including *B4galt1*, *Angpt14*, *Ace*, *Klf4*, *Egf*, *Tgfb2*, *Wnt4* and *Sox10* are simultaneously involved epithelial cell development, angiogenesis and anatomical structure morphogenesis, and the common largely upregulated DEGs *Dnase2b* and *Kcnip3* (upregulated by 7 ~ tenfold) were involved in cell death [15, 16], thus they might be the key candidate genes that mediate androgen deprivation-induced SMG degeneration and dysfunction. In support of our conclusions, of these key candidate genes, *Sox10*, *Wnt4*, *Egf* and *Tgfb2* are all well-known modulators of SMG development and morphogenesis, as demonstrated previously [19–21]. Definitively resolving the mechanism underlying androgen deprivation-induced SMG degeneration and dysfunction, requires experimental modulation of these identified candidate genes, as well as omics data to define the very early events after castration.

Through both histological and transcriptome clustering analysis, our results clearly shown that there existed a differential degeneration response of SMG between the two different methods of castration, that is surgical castration caused a more severe degeneration of SMG than immunocastration. To understand the underlying mechanisms, we analysed the transcriptome data using three different strategies. Firstly, directly functional enrichment analysis of the DEGs between IM and ORC indicated hyperfunction of ribosome and mitochondria, but lower vesicle transport and secretion of SMG in ORC than in IM. Further gene set enrichment analysis (GSEA) using the whole expressed genes also highlighted enhanced translation and oxidative phosphorylation but declined vesicle transport in ORC in relative to either IM or EM (Fig. 8). Thirdly, we isolated the genes whose expressions were significantly changed by ORC but not by IM, which we refer to as the maintained DEGs (mDEGs). The mDEGs are directly relevant to the differential degeneration response of SMG between ORC and IM. Very interestingly, we found more than half (66%) of the DEGs between ORC and EM are mDEGs, and functional enrichment analysis of these mDEGs also highlighted hyperfunction of ribosome and mitochondrion of SMG in ORC rats. All these results commonly demonstrated enhanced protein translation and oxidative phosphorylation of SMG in ORC than in IM. Enhanced protein translation and lower vesicle transport are a typical sign of endoplasmic reticulum (ER) stress [22], which has been demonstrated to be widely linked to cell apoptosis, tissue degeneration, organ damage and disease pathogenesis [22, 23]. But, one caveat to note is that protein translation can appear differentially regulated due to subtle biases in the RNA-seq technique [24]. While, enhanced oxidative phosphorylation usually leads to generation of much reactive oxgen species, thus causing oxidative stress [25]. Both cell apoptosis and oxidative stress have already been demonstrated to be the main causes of degeneration and dysfunction of SMG in postmenopausal women [3, 5]. Therefore, ER stress and oxidative stress secondary to hyperfunction of ribosome and mitochondrion may exacerbate cell death and tissue degeneration of SMG in ORC rats. This may provide a reasonable explanation as to why IM had less extent of SMG degeneration than ORC. To the best of our knowledge, this is the first study to suggest that deprivation of androgens may induce ER stress and thus cause and exacerbate SMG degeneration. Through integrated network analysis, we obtained 27 genes (Fig. 7C) which were mDEGs and also DEGs between ORC and IM. Of these 27 genes, most genes are involved ribosome and mitochondrion, and partial genes are also involved in cell proliferation, cell death, cell junction and lipid metabolism (Fig. 9B and C). We suggest these 27 genes are the candidate mediators of the differential SMG degeneration response between ORC and IM. But, further validations are still warranted.

Besides, we also noted that there was an increase in lipid vacuole infiltration in SMG following ORC. SMG from IM also accumulated lipid vacuoles but it accumulated less than in ORC. In accordance with this phenotype difference, two important lipogenesis regulated genes, *Scap* and *St6galnac2* were selectively upregulated in SMG by ORC but not by IM. *Scap*, encoding sterol regulatory element binding protein (SREBP) cleavage activating protein (SCAP), is a key regulator of SREBP maturation. SCAP induces translocation of SREBP from the endoplasmic reticulum to the Golgi apparatus, allowing it to regulate cellular triglyceride and cholesterol biosynthesis and deposition [26]. *St6galnac2* has been identified to be involved in ferroptosis [27]. Ferroptosis, which is a form of regulated cell death that is dependent on iron and reactive oxygen species, has been reported to be an important cause of SMG degeneration in ovariectomized rodents and postmenopausal women [5]. In agreement with our findings, previous studies also reported that the deterioration of salivary gland structure in ovariectomized rodents or postmenopausal women is accompanied by an increase in lipid droplet infiltration in the salivary glands [2, 5, 28]. It is well known that deficiency of sex steroids usually causes fat ectopic deposition [29]. Circulating sex steroids drop immediately after surgical castration, but maintain high in GnRH vaccinated males until the booster vaccination [12, 13, 30]. Thus, the difference in the nature and timing of suppressed sex hormones between the two different methods of castration may be an important reason to cause less lipid droplet deposition and in turn less extent of SMG degeneration in IM than in ORC as well.

## Conclusion

In this study, we took the advantage of using two different methods of castration to assess the effects of androgen deprivation on histology and physiology of SMG in male rats. Our results clearly demonstrated that deprivation of androgens caused degeneration and dysfunction of SMG, which was associated with disrupted epithelial cell development, angiogenesis, anatomical structure morphogenesis and enhanced cell death. We also found that surgical castration caused more severe SMG degeneration than immunocastration, which may be due to the selectively hyperfunction of ribosome and mitochondrion and more lipid droplet infiltration in SMG. Our findings provide novel insights into the molecular bases of differential degeneration response of SMG between immunocastration and surgical castration, and the mechanisms associated with sex hormone deprivation-induced degeneration of salivary glands.

## Materials and methods

### GnRH vaccine formulation

The GnRH vaccine was prepared exactly as our previous descriptions [30]. Briefly, it consisted of a GnRH-tandem peptide, modified with D-lysine replacing glycine at position 6 of the decapeptide (TDK), conjugated to ovalbumin (OVA), and emulsified in Specol adjuvant (ID-Lelystad formula).

#### Animals and treatment

Twenty-seven adult male Sprague–Dawley (SD) rats were purchased from the HuaXi Laboratory Animal Center of Sichuan University and randomly allocated to one of three groups. Briefly, 9 rats were given the GnRH vaccine, 9 rats underwent bilateral orchiectomy (ORC; at 6 wk of age), and 9 entire males (EM) were given placebo injections (saline) and served as controls. These rats were housed in group cages (three per cage), given ad libitum access to a commercial diet and tap water in a controlled environment with temperature of 21 ± 1 ^0^C, a relative humidity of 50–60% and a 12 h light/12 h dark cycle.

Immunized rats (IM) received 100 ug GnRH peptide equivalent of conjugate at 9 wk of age with a booster vaccination (same dose) given 4 wk later. The vaccine was given im in both hind legs (0.5 mL/leg). All rats were decapitated 7 wk after the booster immunization.

### Samples collection

At 20 wk of age, all rats were anaesthetized with ether and then decapitated. The blood samples (1.5–2.0 mL) were collected, centrifuged at 2000 × g for 15 min at 4 ^0^C and sera were stored at -20 ^0^C pending analysis of hormone concentrations.

After decapitation, the SMG was immediately isolated and weighed. Each SMG was divided into three portions. For RNA-seq, SMG samples from three individual rats were pooled together as a single biological replicate, and three biological replicates (*n* = 3) in each group were used. The remaining one portion from each rat was fixed in Bouin’s solution for histology analysis, and the last portion was frozen in liquid nitrogen and then stored at -80 ^0^C for gene expression analysis. Both testes were excised, dissected free of epididymides, and then weighed as a pair.

### RNA sequencing and data analysis

RNA was extracted from the pooled SMG samples using TRIzol (Invitrogen, Carlsbad, CA, USA). The quality of the total RNA was checked using the Agilent 2100 Bioanalyzer system (Santa Clara, CA, USA). A total amount of 1 μg RNA per sample with RNA integrity numbers (RINs) of 8.5 or greater was used as input material for the RNA sample preparations. Sequencing libraries were generated using NEBNext® UltraTM RNA Library Prep Kit for Illumina® (NEB, USA) following manufacturer’s recommendations. Briefly, mRNA was extracted from total RNA using oligo (dT) magnetic beads and sheared into short fragments of about 200 bases. These fragmented mRNAs were then used as templates for cDNA synthesis. The cDNAs were then PCR amplified to complete the library. The cDNA library was sequenced on a 50-cycle single end run in HiSeq 2500 (Illumina) by Novogene Co., Ltd (Beijing, China). Raw RNA-Seq reads were processed through in-house perl scripts. Clean reads were obtained by removing reads containing low quality reads (> 50% of bases with a quantity score Q-phred ≤ 20), adaptor sequences and reads containing ploy-N from raw reads, and mapped to the rat genome (Rnor 6.0) using Hisat2 software. The gene expression level was then calculated using the reads per kilo bases per million reads (RPKM) method. Pairwise comparisons between groups were performed using the DESeq2 R package (1.16.1). The resulting P-values were adjusted using the Benjamini and Hochberg’s approach for controlling the false discovery rate. Genes with an adjusted *P* value (q value) < 0.05 were assigned as differentially expressed (DEGs). Hierarchical cluster analysis was conducted to assemble genes with similar expression patterns across groups using Cluster 3.0 software. After calculation of the z-score for each gene, gene clustering was performed with an average linkage method with the euclidean distance.

### Functional enrichment analysis

For Gene Ontology (GO) and Kyoto Encyclopedia of Genes and Genomes (KEGG) pathway enrichment analyses of DEGs, the Database for Annotation, Visualization and Integrated Discovery (DAVID) Bioinformatics Resources (v6.8) was used. The genes that were detected in SMG in the experiment were input as the background gene list. In all tests, *P* values were calculated using the Benjamini-corrected modified Fisher’s exact test and *P* ≤ 0.05 or FDR < 0.25 was taken as a threshold of significance. The bubble figures for gene functional enrichment analysis results was plotted with RStudio software using the code (Supplemental file 5).

Gene Set Enrichment Analysis (GSEA) was performed using GSEA v4.0.3 software. Genes including all DEGs and non-DEGs were pre-ranked based on the -log10 (p-value) multiplied by the sign of gene log2 Fold Change such that the up-regulated genes had positive scores and down-regulated had negative scores. This application scores a sorted list of genes with respect to their enrichment of selected functional categories (Gene Ontology [GO], Kyoto Encyclopedia of Genes and Genomes[KEGG], Reactome and Biocarta). Terms annotating more than 500 or less than 5 genes were discarded. The significance of the enrichment score was assessed using 1000 permutations and default of Benjamini and Hochberg's false discovery rate (FDR) < 0.25 was considered significant.

### Serum hormones assays

Serum testosterone concentrations were quantified by commercial RIA Kits (Immunotech, Marseille, France). The sensitivity of the assay was 0.025 ng/mL, with intra- and inter-assay CVs of 10 and 15%, respectively.

Serum concentrations of LH and FSH were measured by rat-specific RIA kits (DRG International, Marburg, Germany). For both assays, sensitivities were 1.0 mIU/mL and the intra- and inter-assay CVs were 10 and 15%, respectively.

### Submandibular gland histology

SMG from each rat was collected and fixed in 10% buffered formalin for 48 h, and then paraffin embedded. Then, the SMG was serially sectioned at 5-μm thickness, and were subjected to standard H&E staining for morphological observation. The quantity of serous or seromucous acini and striated ducts were individually counted as described previously and finally evaluated in percentage [6]. The number of lipid vacuoles in each section was also counted.

### Data validation by real-time quantitative PCR (RT-qPCR)

Total RNA was isolated from SMG according to manufacturer’s instructions (Invitrogen Co., Carlsbad, CA, USA). Quantitative and qualitative analyses of isolated RNA were assessed from the ratio of absorbance at 260 and 280 nm and agarose gel electrophoresis. A total of 1000 ng RNA was converted into first-strand cDNA using a PrimeScript® RT reagent kit with gDNA Eraser (TaKaRa Bio, Co., LTD, Dalian, China). Quantitative real-time PCR was done on a CFX96 Real Time PCR detection system (Bio Rad, USA). The PCR reaction contained 1μL cDNA, 500 nmol/L each of forward and reverse primers, and 2XSYBR® premix TaqTM (TaKaRa Bio Co., Ltd.). Primer sequences of target and reference genes are shown (Supplemental file 1). The PCR cycling conditions were: initial denaturation at 95 °C (1 min), following by 40 cycles of denaturation at 95 °C (5 s), annealing at 60 °C (25 s) and a final melting curve analysis (to monitor PCR product purity). A reference house-keeping gene (*β-actin*) was measured for each sample. The fold change of mRNA in the treatment group in relative to control group (EM) was determined by 2^−ΔΔCt^.

### Statistical analyses

Statistical analysis was performed using GraphPad Prism 9 (La Jolla, CA) software. Comparisons among groups were carried out via one-way ANOVA followed by Turkey’s test. For analysis of effect of treatment in repeated-measures (i.e., body weight), two-way ANOVA followed by Sidak's multiple comparisons test was used. All values were expressed as mean ± SEM and statistical significance was defined as *P* < 0.05.

## Supplementary Information

**Additional file 1. **qPCR Primer sequences for tissue genes.

**Additional file 2.** DEGs betweentreatment groups and mDEGs.

**Additional file 3. **Common DEGs regulated by both ORC and IM.

**Additional file 4. **Functional enrichment analysis of DEGs between groups and mDEGs using DAVID) Bioinformatics Resources.

**Additional file 5. **The code to plot the bubble figures for gene functional enrichment analysis results from DAVID. 

**Additional file 6.  **Supplementary statement docx.

## Data Availability

All data generated or analysed during this study are included in this published article and its supplementary information files. The RNA sequencing raw data generated during the current study was available in the GSA database (https://bigd.big.ac.cn/gsa/browse; Accession: CRA005783).

## Abbreviations

SMG: Submaxillary gland
EM: Entire males
IM: Immunocastration
ORC: Bilateral orchiectomy
LH: Luteinizing hormone
FSH: Follicle-stimulating hormone
GnRH: Gonadotropin-releasing hormone
RT-qPCR: Real-time quantitative PCR
FPKM: Fragments per kilobase of transcripts per million
GO: Gene ontology
KEGG: Kyoto Encyclopedia of Genes and Genomes
BP: Biological process
MF: Molecular Function
CC: Cellular Component
GSEA: Gene Set Enrichment Analysis

## References

[1] Porcheri C, Mitsiadis TA (2019). Physiology, Pathology and Regeneration of Salivary Glands. Cells.

[2] Toan NK, Ahn SG (2021). Aging-Related Metabolic Dysfunction in the Salivary Gland: A Review of the Literature. Int J Mol Sci.

[3] Pedersen A, Sørensen CE, Proctor GB, Carpenter GH (2018). Salivary functions in mastication, taste and textural perception, swallowing and initial digestion. Oral Dis.

[4] Shafer WG, Muhler JC (1953). Effect of gonadectomy and sex hormones on the structure of the rat salivary glands. J Dent Res.

[5] Kwon HK, Kim JM, Shin SC, Sung ES, Kim HS, Park GC (2020). The mechanism of submandibular gland dysfunction after menopause may be associated with the ferroptosis. Aging (Albany NY).

[6] Carvalho VD, Silveira VÁ, do Prado RF, Carvalho YR (2011). Effect of estrogen therapy, soy isoflavones, and the combination therapy on the submandibular gland of ovariectomized rats. Pathol Res Pract.

[7] Streckfus CF, Baur U, Brown LJ, Bacal C, Metter J, Nick T (1998). Effects of estrogen status and aging on salivary flow rates in healthy Caucasian women. Gerontology.

[8] Lago ML, de Oliveira AE, Lopes FF, Ferreira EB, Rodrigues VP, Brito LM (2015). The influence of hormone replacement therapy on the salivary flow of post-menopausal women. Gynecol Endocrinol.

[9] Thompson DL (2000). Immunization against GnRH in male species (comparative aspects). Anim Reprod Sci.

[10] Junco JA, Rodríguez R, Fuentes F, Baladrón I, Castro MD, Calzada L (2019). Safety and Therapeutic Profile of a GnRH-Based Vaccine Candidate Directed to Prostate Cancer. A 10-Year Follow-Up of Patients Vaccinated With Heberprovac. Front Oncol.

[11] Han X, Meng F, Cao X, Du X, Bu G, Kong F (2021). FSH promotes fat accumulation by activating PPARγ signaling in surgically castrated, but not immunocastrated, male pigs. Theriogenology.

[12] Han X, Gu L, Xia C, Feng J, Cao X, Du X (2015). Effect of immunization against GnRH on hypothalamic and testicular function in rams. Theriogenology.

[13] Han XF, Cao XH, Tang J, Du XG, Zeng XY (2013). Active immunization against GnRH reduces the synthesis of GnRH in male rats. Theriogenology.

[14] Ramsköld D, Wang ET, Burge CB, Sandberg R (2009). An abundance of ubiquitously expressed genes revealed by tissue transcriptome sequence data. PLoS Comput Biol..

[15] Saito Y, Hikita H, Nozaki Y, Kai Y, Makino Y, Nakabori T (2019). DNase II activated by the mitochondrial apoptotic pathway regulates RIP1-dependent non-apoptotic hepatocyte death via the TLR9/IFN-β signaling pathway. Cell Death Differ.

[16] Lilliehook C, Chan S, Choi EK, Zaidi NF, Wasco W, Mattson MP (2002). Calsenilin enhances apoptosis by altering endoplasmic reticulum calcium signaling. Mol Cell Neurosci.

[17] Forsblad-d'Elia H, Carlsten H, Labrie F, Konttinen YT, Ohlsson C (2009). Low serum levels of sex steroids are associated with disease characteristics in primary Sjogren's syndrome; supplementation with dehydroepiandrosterone restores the concentrations. J Clin Endocrinol Metab.

[18] Lents MP, Barbosa LP, Santana ALA, Pinheiro EEG, Mugabe LC, Biscarde CEA (2018). Immunocastration of goats using anti-gonadotrophin releasing hormone vaccine. Theriogenology.

[19] Aure MH, Symonds JM, Mays JW, Hoffman MP (2019). Epithelial Cell Lineage and Signaling in Murine Salivary Glands. J Dent Res.

[20] Jaskoll T, Melnick M (1999). Submandibular gland morphogenesis: stage-specific expression of TGF-alpha/EGF, IGF, TGF-beta, TNF, and IL-6 signal transduction in normal embryonic mice and the phenotypic effects of TGF-beta2, TGF-beta3, and EGF-r null mutations. Anat Rec.

[21] Mizukoshi K, Koyama N, Hayashi T, Zheng L, Matsuura S, Kashimata M (2016). Shh/Ptch and EGF/ErbB cooperatively regulate branching morphogenesis of fetal mouse submandibular glands. Dev Biol.

[22] Han J, Back SH, Hur J, Lin YH, Gildersleeve R, Shan J (2013). ER-stress-induced transcriptional regulation increases protein synthesis leading to cell death. Nat Cell Biol.

[23] Lin JH, Walter P, Yen TS (2008). Endoplasmic reticulum stress in disease pathogenesis. Annu Rev Pathol.

[24] Mandelboum S, Manber Z, Elroy-Stein O, Elkon R (2019). Recurrent functional misinterpretation of RNA-seq data caused by sample-specific gene length bias. PLoS Biol..

[25] Kudryavtseva AV, Krasnov GS, Dmitriev AA, Alekseev BY, Kardymon OL, Sadritdinova AF (2016). Mitochondrial dysfunction and oxidative stress in aging and cancer. Oncotarget.

[26] Lee SH, Lee JH, Im SS (2020). The cellular function of SCAP in metabolic signaling. Exp Mol Med.

[27] Tang R, Hua J, Xu J, Liang C, Meng Q, Liu J (2020). The role of ferroptosis regulators in the prognosis, immune activity and gemcitabine resistance of pancreatic cancer. Ann Transl Med.

[28] Scott J (1977). Quantitative age changes in the histological structure of human submandibular salivary glands. Arch Oral Biol.

[29] Quinn MA, Xu X, Ronfani M, Cidlowski JA (2018). Estrogen Deficiency Promotes Hepatic Steatosis via a Glucocorticoid Receptor-Dependent Mechanism in Mice. Cell Rep.

[30] Han XF, Li JL, Zhou YQ, Ren XH, Liu GC, Cao XH (2016). Active immunization with GnRH-tandem-dimer peptide in young male rats reduces serum reproductive hormone concentrations, testicular development and spermatogenesis. Asian J Androl.

[31] Percie du Sert N, Ahluwalia A, Alam S, Avey MT, Baker M, Browne WJ (2020). Reporting animal research: Explanation and elaboration for the ARRIVE guidelines 2.0.. PLoS Biol.

