# Supplementary material for: RNA-seq coupling two different methods of castration reveals new insights into androgen deficiency-caused degeneration of submaxillary gland in male Sprague Dawley rats
Source: BMC Genomics. 2022 Apr 7;23:279. doi: 10.1186/s12864-022-08521-9 (PMC8991617; doi:10.1186/s12864-022-08521-9)
Supplement: Supplementary file 1 — Additional file 1. qPCR Primer sequences for tissue genes. [file 12864_2022_8521_MOESM1_ESM.doc]

**Table S1**

qPCR Primer sequences for tissue genes

| **Gene** | **Genbank accession no.** | **Primer sequence (5’-3’)** | **Amplification length (bp)** |
| --- | --- | --- | --- |
| B4galt1 | NM_053287.1 | F: CGCTTTGTGTTCAGTGATGTGG  R: AACGTAAGGCAGGCTAAACC | 123 |
| Angptl4 | NM_199115.2 | F: GCCGCTACTATCCACTAC  R: CCTGTTGCTCTGACTGTT | 282 |
| Ace | NM_012544.1 | F: CACCGGCAAGGTCTGCTT  R: CTTGGCATAGTTTCGTGAGGAA | 97 |
| Klf4 | NM_053713.1 | F: GAGAGGAACTCTCTCACATGAAGC  R: AAGGATAAAGTCTAGGTCCAGGAGA | 185 |
| Egf | NM_012842.2 | F: ACTATAACGGTGGCTCCATCC  R: CAGTGTGTTTGTCGGCTATCC | 135 |
| Tgfb2 | NM_031131.2 | F: TTGGATGCCGCCTATTGCTT  R: TACAGGCTGAGGACTTTGGTG | 191 |
| Wnt4 | NM_053402.2 | F: CTCGTCTTCGCCGTGTTCTC  R: GCACTGAGTCCATCACCTCG | 166 |
| Sox10 | NM_019193.3 | F: AGCCCAGGTGAAGACAGAGA  R: CCCCTCTAAGGTCGGGATAG | 348 |
| Ccnd1 | NM_171992.5 | F: GCCCTCCGTTTCTTACTTC  R: AGACCTCCTCTTCGCACTTC | 116 |
| Dnase2b | NM_021664.2 | F: ACAGGGATGGGGCTGGATTA  R: GCAGGGACAGCGTCATTGTA | 176 |
| Kcnip3 | NM_032462.2 | F: GATGCAGAGGACCAAGGAAGCA  R: CTCTTGCTCAGCGGTATGCG | 81 |
| *β-actin* | NM_031144 | F: CACAGCTGAGAGGGAAAT  R: TCAGCAATGCCTGGGTAC | 155 |

*Abbreviations: B4galt1*, beta-1,4-galactosyltransferase 1; *Angptl4*, angiopoietin-like 4; *Ace*, angiotensin I converting enzyme; *Klf4*, Kruppel like factor 4; *Egf*, epidermal growth factor; *Tgfb2*, transforming growth factor, beta 2; *Wnt4*, Wnt family member 4; *Sox10*, SRY-box transcription factor 10; *Ccnd1*, cyclin D1; *Dnase2b*, deoxyribonuclease 2 beta; *Kcnip3*, potassium voltage-gated channel interacting protein 3;*β-actin*, actin beta.
