# Supplementary material for: RNA-seq coupling two different methods of castration reveals new insights into androgen deficiency-caused degeneration of submaxillary gland in male Sprague Dawley rats
Source: BMC Genomics. 2022 Apr 7;23:279. doi: 10.1186/s12864-022-08521-9 (PMC8991617; doi:10.1186/s12864-022-08521-9)
Supplement: Supplementary file 5 — Additional file 5. The code to plot the bubble figures for gene functional enrichment analysis results from DAVID. [file 12864_2022_8521_MOESM5_ESM.doc]

library(ggplot2)

goinput <- read.csv("Data.csv",header=TRUE,row.names=1,check.names = FALSE)

x=goinput$GeneRatio

y=factor(goinput$Pathway,levels = goinput$Pathway)

p = ggplot(goinput,aes(x,y))

p1 = p + geom_point(aes(size=Count,color=FDR))+scale_color_gradient(low = "red",high = "blue",limits=c(0.00,0.25))

p2 = p1 + labs(color=expression(FDR),size="Count",x="GeneRatio",y="")

p3 = p2 +theme_bw()+theme(axis.title=element_text(size=14,face="bold"))

p4 = p3 +theme_bw()+theme(axis.text.y=element_text(size=11))

ggsave("out.pdf",width=6,height=8)
