# Supplementary material for: RNA-seq coupling two different methods of castration reveals new insights into androgen deficiency-caused degeneration of submaxillary gland in male Sprague Dawley rats
Source: BMC Genomics. 2022 Apr 7;23:279. doi: 10.1186/s12864-022-08521-9 (PMC8991617; doi:10.1186/s12864-022-08521-9)
Supplement: Supplementary file 6 — Additional file 6. Supplementary statement docx. [file 12864_2022_8521_MOESM6_ESM.docx]

Supplemental file 1: qPCR Primer sequences for tissue genes.

Supplemental file 2: DEGs between treatment groups and mDEGs.

Supplemental file 3: Common DEGs regulated by both ORC and IM.

Supplemental file 4: Functional enrichment analysis of DEGs between groups and mDEGs using DAVID) Bioinformatics Resources.

Supplemental file 5: The code to plot the bubble figures for gene functional enrichment analysis results from DAVID.
